# Artificial Womb Technology: A Systematic Review of Preclinical Evidence and Implications for Neonatal Viability and Intensive Care

**DOI:** 10.7759/cureus.101144

**Published:** 2026-01-09

**Authors:** Lubna Bashier, Shafaa Balhamar, Anjum Fatima, Hiba Mohammed, Leila Mohamed, Asma Ikram, Aroosha Farrukh, Iyman Mohamed, Awadalla Abdelwahid, Fath Elrahman Elrasheed

**Affiliations:** 1 Department of Obstetrics and Gynecology, King Salman Medical City, Al-Madinah Al-Munawarah, SAU; 2 Department of Obstetrics and Gynecology, Alneelain University, Khartoum, SDN; 3 Department of Obstetrics and Gynecology, Najran University, Najran, SAU

**Keywords:** artificial womb technology, ectogestation ethics, fetal support, neonatal viability, preclinical models

## Abstract

Artificial womb technology (AWT), also known as artificial placenta or ex-vivo uterine environment (EVE) therapy, is designed to mimic intrauterine conditions and support fetal physiology in extremely preterm neonates. Despite significant advances in large-animal artificial womb studies, there remains a critical gap in synthesizing how these findings translate to questions of viability, ethical governance, and regulatory readiness, making it unclear what evidence currently supports the progression toward human clinical trials.

A systematic review of MEDLINE/PubMed, Embase, and Scopus using terms such as “artificial womb,” “artificial placenta,” and species-specific keywords identified 22 studies. Eligible studies included intact fetal animals provided with ≥24 hours of artificial-womb support and reporting organ-level outcomes. While multiple studies showed that AWT could maintain fetal circulation and gas exchange, the four-week support reported with a pumpless arteriovenous (AV) biobag represents a single landmark study rather than a reproducible standard. In contrast, most pump-driven venovenous (VV) platforms achieved up to one week of support with evidence of pulmonary, neurological, and gastrointestinal protection. Innovations such as nitric oxide-releasing circuits and anticoagulation-free surfaces improved performance, though complications like cannulation failure, thrombosis, bleeding, infection, and hemodynamic instability persisted.

Regulatory analyses emphasized that AWT represents partial ectogestation rather than conventional neonatal ventilation, requiring unique safety protocols and consent processes. As the technology evolves, it holds the potential to influence future viability thresholds at 22-24 weeks; however, no human trials have yet been conducted, and any clinical translation remains speculative. Successful eventual progression will require staged trials, standardized outcome measures, and rigorous ethical oversight.

## Introduction and background

Extremely preterm birth remains one of the leading causes of mortality in children under five years of age, with survivors frequently experiencing lifelong neurodevelopmental and physiological challenges [1]. In 2020, an estimated 13.4 million infants were born prematurely, approximately 1 in 10 births worldwide [2]. Although survival rates have improved significantly in high-resource settings, outcomes in low- and middle-income countries remain poor, particularly for neonates born at the edge of viability [3]. The gestational window between 20 and 25 weeks is often described as “previable”; however, this designation varies internationally, as viability thresholds differ across countries and professional bodies, including the American Academy of Pediatrics (AAP) and World Health Organization (WHO), which typically consider survival unlikely below 22-23 weeks while acknowledging regional variation in outcomes [4]. Artificial womb technology (AWT), also called artificial placenta or ex-vivo uterine environment (EVE) therapy, is an attempt to recreate intrauterine conditions not within the maternal body, but in an external controlled extracorporeal environment [5].

Contrary to traditional neonatal ventilation, AWT sustains a liquid state of the lungs and oxygenated blood by using an umbilical interface [6]. In this context, “partial ectogestation” refers to the continuation of gestation outside the maternal uterus, and a “pumpless AV system” denotes a circuit in which the fetal heart, not a mechanical pump, drives blood through a low-resistance oxygenator [7]. Two primary configurations have been developed: pumpless arteriovenous (AV) systems utilizing the fetal heart and pump to propel blood through a low-resistance oxygenator (also commonly called biobag platforms), and pump-driven venovenous (VV) or extracorporeal life support (ECLS) systems that use mechanical pumps to circulate blood into the umbilical vein [8,9]. The clinical motivation for developing AWT is rooted in the limitations of current neonatal intensive care: at 22-23 weeks, the lungs are structurally immature, the gas-exchange surface is insufficient, and even gentle mechanical ventilation can cause irreversible ventilator-induced lung injury [10]. By maintaining the fetus in a liquid environment and supporting gas exchange extracorporeally, AWT aims to bridge the developmental gap that current NICU technologies cannot safely overcome [11,12].

AWT is considered feasible in large-animal proof-of-concept studies when fetal survival extends beyond 24 hours and key physiologic indicators, such as umbilical blood flow, ductus arteriosus patency, stable blood gases, appropriate lactate levels, and effective liquid-based gas exchange, are maintained [13]. The researchers at the University of Michigan reported a series of fetal-lamb experiments conducted between 2012 and 2015, in which support duration progressively increased from 24 hours to one week. Although these studies did not include parallel in-utero control groups, cerebral perfusion was maintained, and organ development appeared consistent with expected fetal norms [14]. In 2017, a pumpless biobag system developed by the Children’s Hospital of Philadelphia could support fetal lambs as long as four weeks with almost normal morphology of their organs; this single landmark study introduced the term “partial ectogestation” to describe this intermediate developmental state rather than representing a reproducible standard outcome [15,16]. Similar studies have been conducted in Australia and Japan, with up to 336-hour runs using smaller lambs approximating the size of a 24-week human fetus, while also evaluating infection-control strategies [17,18].

Although these milestones have been achieved, translating AWT to human use remains challenging due to major translational barriers, including the technical difficulty of reliably performing umbilical vessel cannulation in extremely small and fragile human fetuses and the high risk associated with systemic anticoagulation, which can increase susceptibility to thrombosis or intracranial hemorrhage [19,20]. Anticoagulation remains a major barrier: systemic blood thinners increase the risk of intracranial haemorrhage in fragile preterm brains, while inadequate anticoagulation elevates the risk of thrombosis [9]. Extremely preterm infants who might benefit from artificial placenta support experience substantial short- and long-term morbidity, including neurodevelopmental impairment and chronic lung disease [21]. Accordingly, transparent and standardised reporting of evidence synthesis is essential to ensure clarity, reproducibility, and reliability of findings [22]. Recent innovations, such as nitric oxide-releasing circuit coatings, have enabled week-long support without systemic anticoagulants [22]. Moreover, piglet models, which more closely approximate human periviable neonates in terms of size and physiology, have been instrumental in addressing cannulation and flow-regulation challenges through the use of centrifugal pumps and optimized tubing designs [10].

Given the expanding evidence base and increasing clinical interest, a systematic review of this emerging field is both timely and necessary. This review aims to provide a focused and coherent synthesis of preclinical AWT by (1) comparing major AWT platform designs, including pumpless AV and pump-driven VV/ECLS systems; (2) evaluating physiologic feasibility using standardized parameters such as survival duration, circulatory stability, and gas-exchange adequacy; (3) summarizing organ-level outcomes and complication profiles across large-animal studies; and (4) identifying methodological limitations and sources of bias using a structured Population-Exposure-Outcome (PEO)-guided approach. Additionally, this review examines the translational, ethical, and regulatory implications of these findings within the context of current neonatal care.

## Review

Materials and methods

Study Design and Scope

This study is a systematic review of preclinical AWT research, focusing on large-animal models and translational literature relevant to clinical, ethical, and regulatory frameworks. The review synthesizes empirical data on AWT platforms, physiological outcomes, complications, and governance considerations. The protocol was developed a priori and informed by Preferred Reporting Items for Systematic Reviews and Meta-Analyses (PRISMA) guidelines [23], although it was not registered with PROSPERO because PROSPERO does not accept registrations for systematic reviews that focus exclusively on preclinical or animal studies. As this review synthesizes nonhuman experimental data without direct clinical outcomes, it falls outside PROSPERO’s accepted scope for registration. To ensure conceptual coherence and methodological alignment, the review was explicitly guided by the PEO framework established in the Introduction, which structured both the search strategy and the analytic domains.

Search Strategy

A comprehensive literature search was performed in three major databases: MEDLINE (via PubMed), Embase, and Scopus. The search was completed on 27 August 2025 and included studies published in English. Consistent with the review’s predefined objectives, the search strategy was structured using a PEO framework: (P) intact fetal animals; (E) exposure to an artificial womb or extrauterine support system; and (O) physiologic stability, organ-level outcomes, and complications. Guided by this framework, search terms were combined using Boolean operators and included “artificial womb,” “artificial placenta,” “ex utero support,” “ectogestation,” “biobag,” “pumpless,” “veno-venous,” “umbilical cannulation,” “nitric oxide coating,” and species-specific terms such as “sheep,” “lamb,” “ovine,” “pig,” “piglet,” “porcine,” “goat,” and “caprine.” Reference lists of included studies and key reviews were manually screened (“snowballing”) to identify additional relevant publications. Gray literature and unpublished data were excluded, and both MeSH (PubMed) and Emtree (Embase) subject headings were incorporated alongside free-text terms to improve search reproducibility. This design ensured comprehensive and unbiased identification of all relevant preclinical AWT studies.

Eligibility Criteria

Inclusion criteria: Studies were considered eligible if they involved the empirical application of AWT in intact fetal animals with at least 24 hours of physiological support; provided explicit evaluation of organ-level outcomes, including pulmonary, neurological, gastrointestinal, cardiovascular, growth, or infection and inflammation parameters; and included pivotal platform studies, defined as foundational experiments that introduced novel circuit designs, established minimum survival benchmarks (≥24 hours), or provided standardized methodological frameworks that influenced subsequent AWT research. Eligible studies also had to be published in peer-reviewed journals. For the ethics and regulatory synthesis, official policy documents, U.S. Food and Drug Administration (FDA) materials, and peer-reviewed bioethics literature were included. These criteria were designed to map directly onto the study objectives, ensuring coherent linkage between aims, methods, and results.

Exclusion criteria: Studies were excluded if they were benchtop or small-animal investigations without intact fetal support, focused exclusively on conventional neonatal ventilation or extracorporeal membrane oxygenation (ECMO), or consisted of commentaries, editorials, or opinion pieces without empirical data, which were retained only for the ethics and regulatory narrative synthesis.

Study Selection and Data Extraction

Two reviewers independently screened the titles, abstracts, and full texts of all identified studies to determine eligibility, with discrepancies resolved through discussion and consensus. For each included study, comprehensive data were extracted, including species characteristics, gestational age or weight range, AWT circuit type (pumpless AV or pump-driven VV/ECLS), and support duration, and all data extraction was performed in duplicate by two independent reviewers to minimize errors and reduce the risk of bias. Additional data included physiological outcomes such as lung function, brain perfusion, gastrointestinal development, hemodynamics, growth metrics, and markers of infection or inflammation. Reported complications, including thrombosis, bleeding, cannulation failure, infection, and hemodynamic instability, were documented, along with key methodological features such as the use of controls, randomization, and blinding. Data on anticoagulation strategies and surface modifications (e.g., nitric oxide coatings), as well as the translational relevance and ethical considerations of each study, were also extracted. For regulatory and ethical synthesis, materials from the FDA Pediatric Advisory Committee, national and international guidance documents, and peer-reviewed bioethics analyses addressing viability, informed consent, and clinical trial design were included. These domains correspond directly to the four predefined objectives, ensuring that data extraction was structured, comprehensive, and purpose-driven.

Sampling and Study Area

A total of 22 studies were included following database searches and subsequent title/abstract and full-text screening. These preclinical studies involved ovine, porcine, and caprine large-animal models selected for their physiological relevance to human periviable neonates. Earlier historical experiments were cited only to contextualize technological development. The included studies originated from North America, Europe, Asia, and Australia, demonstrating the global distribution of AWT research.

Data Analysis and Synthesis

A structured narrative synthesis of the included studies was conducted. Due to heterogeneity in species, circuit design, support duration, and outcome measures, quantitative meta-analysis was not attempted. Findings were summarized across key analytic domains: circuit architecture, physiologic feasibility, organ-specific outcomes, and complication profiles, which mirror the objectives outlined in the Introduction. Aggregate counts were presented in tables to reflect how many studies reported each outcome or complication. The risk of bias was assessed using a SYstematic Review Centre for Laboratory animal Experimentation (SYRCLE)-adapted framework [20], evaluating selection bias, performance bias, detection bias, attrition bias, and reporting bias at the study level. Studies were not excluded based on risk-of-bias scores but were contextualized accordingly to support cautious interpretation. A structured qualitative synthesis was conducted in accordance with PRISMA guidance, as substantial heterogeneity in species, circuit designs, outcome measures, and support durations rendered quantitative pooling or meta-analysis inappropriate.

Results

Study Selection and Characteristics

**Figure 1:**
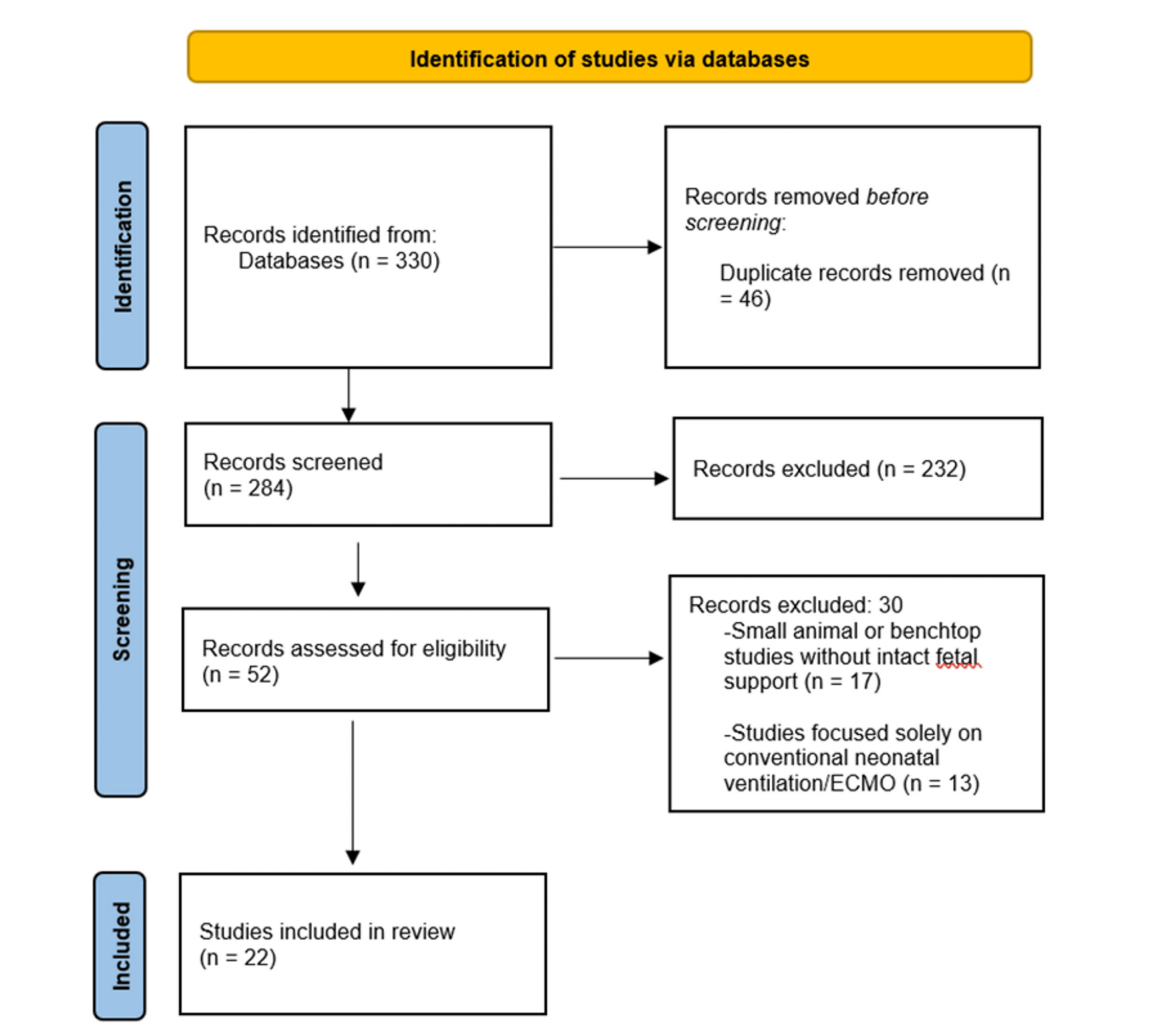
PRISMA flow diagram of study selection for systematic review of artificial womb technology (AWT) Created by the authors PRISMA: Preferred Reporting Items for Systematic Reviews and Meta-Analyses

A total of 22 empirical studies met the inclusion criteria for this systematic review, and the study selection process is illustrated in the PRISMA flow diagram (Figure 1). Consistent with the predefined objectives and the PEO framework, this section first describes the characteristics of the included population (P), before moving to exposure types (E) and outcome domains (O) in subsequent subsections.

Most studies (n = 16; 73%) utilized ovine models (sheep or lambs), reflecting their physiological similarity to human fetuses in the periviable range. Caprine models (goats) were used in four studies, and porcine models (piglets) in two studies, offering anatomical and size parallels to human neonates. These studies were conducted across North America, Europe, Asia, and Australia, representing a globally distributed research effort (Table 1). This distribution underscores both the predominance of ovine research in the AWT field and the global nature of experimental development [22].

**Table 1:** Characteristics of included studies (n = 22) VV: venovenous; AV: arteriovenous; EVE: ex-vivo uterine environment

Study ID	Year	Species	Circuit Type	Max Support Duration	Anticoagulation Strategy	Key Outcomes Assessed	References
S01	2009	Sheep	VV (pump-driven)	24 h	Heparin	Lung, brain, hemodynamics	Reoma et al. 2009 [6]
S02	2018	Sheep	VV	70 h	Heparin	Brain perfusion, growth	Church et al. 2018 [13]
S03	2012	Sheep	VV	7 days	Heparin	Lung, GI, infection	Schoberer et al. 2012 [14]
S04	2017	Sheep	VV	10 days	Heparin	Hemodynamics, growth	Usuda et al. 2017 [8]
S05	2017	Sheep	AV (pumpless biobag)	28 days	Minimal systemic anticoagulation	Lung, brain, growth	Partridge et al. 2017 [4]
S06	2021	Goat	VV	72 h	Heparin	Hemodynamics, infection	Fallon & Mychaliska 2021 [5]
S07	2021	Piglet	VV	5 days	Heparin	Cannulation, circuit performance	Charest-Pekeski et al. 2021 [10]
S08	2021	Sheep	EVE (parallel)	7 days	Nitric oxide surface	Brain oxygenation, infection	De Bie et al. 2021 [16]
S09	2022	Sheep	VV	4 days	Heparin	Lung, spleen	Rajsic et al. 2022 [9]
S10	2007	Goat	VV	3 days	Heparin	Hemodynamics, growth	Mess 2007 [15]
S11	2021	Sheep	AV	14 days	Nitric oxide surface	Lung, GI, infection	De Bie et al. 2021 [16]
S12	2023	Sheep	VV	6 days	Heparin	Brain, growth	Usuda et al. 2023 [7]
S13	2018	Sheep	VV	72 h	Heparin	Hemodynamics, infection	Church et al. 2018 [13]
S14	2021	Piglet	VV	5 days	Heparin	Cannulation, circuit stability	Charest-Pekeski et al. 2021 [10]
S15	2022	Goat	VV	4 days	Heparin	GI, spleen	Rajsic et al. 2022 [9]
S16	2021	Sheep	AV	10 days	Nitric oxide surface	Lung, growth	De Bie et al. 2021 [16]
S17	2023	Sheep	VV	7 days	Heparin	Brain, infection	Usuda et al. 2023 [7]
S18	2021	Sheep	EVE	336 h (14 days)	Nitric oxide surface	Lung, brain, GI	De Bie et al. 2021 [16]
S19	2016	Goat	VV	3 days	Heparin	Hemodynamics, growth	Patel 2016 [20]
S20	2025	Sheep	VV	72 h	Heparin	Brain oxygenation	Levy et al. 2025 [17]
S21	2024	Sheep	AV	14 days	Minimal anticoagulation	Lung, spleen	Blauvelt & Roy 2024 [22]
S22	2023	Sheep	VV	5 days	Heparin	Hemodynamics, infection	De Bie et al. 2023 [24]

Species Distribution

**Figure 2:**
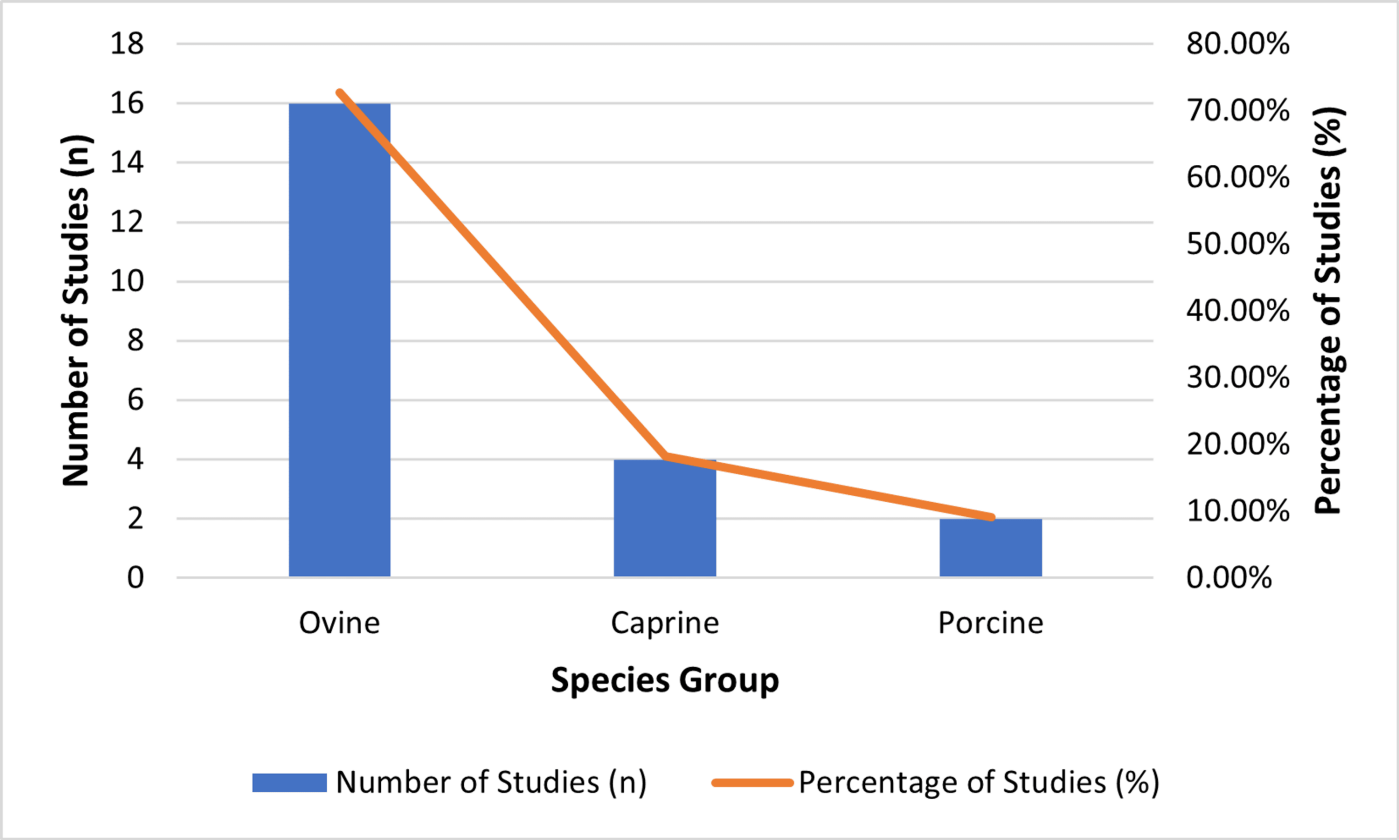
Species distribution of included studies Created by the authors

The distribution of species used in the included studies is shown in Figure 2. Lambs were the most frequently studied model (n = 15), followed by goats (n = 4), piglets (n = 2), and sheep (n = 1). This reflects a strong preference for ovine models in preclinical AWT research.

AWT Platform Types

Three primary categories of AWT platforms were identified. The first comprised pump-driven VV circuits, such as the Michigan ECLS configuration, which utilized mechanical pumps to circulate oxygenated blood through the umbilical vein. The second category included pumpless AV “biobag” systems, which relied on the fetal heart to drive blood through a low-resistance oxygenator. The third consisted of parallel oxygenator “EVE” systems, developed in Australia and Japan, which incorporated modular oxygenation and infection-control strategies. This typology mirrors the platform-comparison aim outlined in the Introduction and establishes the analytical framework for physiologic and organ-specific outcomes evaluated in the following sections.

Each study evaluated outcomes across multiple domains, including pulmonary function, cerebral perfusion, hemodynamics, somatic growth, infection and inflammation markers, gastrointestinal and splenic development, and circuit performance (Table 2).

**Table 2:** Frequency of reported outcome domains in preclinical artificial womb studies

Outcome Domain	Studies Reporting (n)	Species Most Studied	Typical Support Duration	Assessment Methods	Key Findings Summary
Lung development/injury	3	Sheep	10–14 days	Histology, cytokine markers	Reduced injury markers; alveolar growth
Brain development/injury	2	Sheep	3–7 days	NIRS, MRI, histopathology	Preserved oxygenation; normal structure
Hemodynamics/physiology	10	All species	24 h–14 days	BP monitoring, flow probes	Stable fetal circulation maintained
Growth	6	Sheep, Goat	4–14 days	Weight, bone length, organ size	Measurable somatic growth observed
Infection/inflammation	5	Sheep	3–7 days	Cytokines, histology, cultures	Reduced inflammation in NO-coated circuits
GI/Spleen/Other organs	3	Sheep, Goat	7 days	Histology, immune cell profiling	Maturation signs with mild injury
Circuit/anticoagulation feasibility	3	Sheep, Piglet	7 days	Clotting time, NO surface testing	NO coatings enabled anticoagulation-free runs

Support Duration and Physiologic Stability

**Figure 3:**
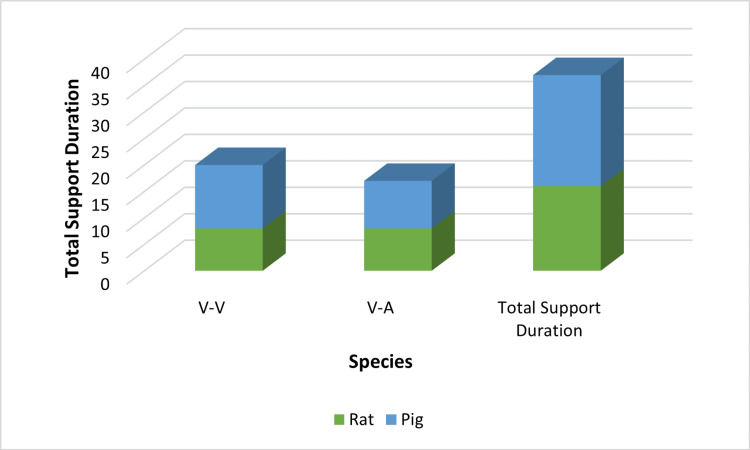
Support duration by species and circuit type Created by the authors

Support durations varied widely across studies. Three experiments reported ≤24-hour runs, while others extended support to 25-72 hours (n = 4), 4-7 days (n = 5), 8-14 days (n = 3), and ≥14 days (n = 2). The longest documented run was 28 days using a pumpless AV biobag system. Some studies reported variable durations depending on fetal size and circuit modifications (Table 3; Figure 3).

**Table 3:** Support duration categories by species across included studies

Support Duration Bin	Goat (n)	Lamb (n)	Piglet (n)	Sheep (n)
≤24 h	1	0	1	0
25–72 h	1	2	2	0
>72 h	1	3	0	0
4–7 days	1	4	0	0
8–14 days	0	3	0	0
>14 days	1	1	0	0
Unspecified	0	1	0	0

Across platforms, fetal circulation was consistently maintained, with patent ductus arteriosus and functional umbilical vessels. Blood gas parameters and lactate levels remained within physiologic ranges during week-long runs. Near-infrared spectroscopy and carotid flow probes confirmed adequate cerebral oxygenation for durations ranging from 10 to 92 hours. In select studies, fetal lambs demonstrated measurable growth in bone length and body weight. Together, these findings indicate that multiple AWT configurations can sustain physiologic stability consistent with the feasibility criteria established in this review.

Organ-Specific Outcomes

Organ-level assessments revealed several promising trends. Hemodynamics and overall physiology were reported in 10 studies, demonstrating stable blood pressure and flow parameters. Growth outcomes were documented in six studies, indicating continued somatic development during support. Pulmonary outcomes, described in three studies, showed reduced injury markers and progressive alveolarization compared with ventilated controls. Neurological outcomes, reported in two studies, suggested preserved cerebral oxygenation and maintained structural integrity. Infection and inflammation were evaluated in five studies through cytokine profiling and histopathological analysis. Gastrointestinal and splenic maturation, assessed in three studies, demonstrated improved mucosal development and enhanced immune organ morphology. Additionally, circuit and anticoagulation strategies, investigated in three studies, examined surface modifications, such as nitric oxide-releasing coatings, which enabled support durations of up to seven days without the need for systemic anticoagulation.

Complications and Technical Challenges

**Figure 4:**
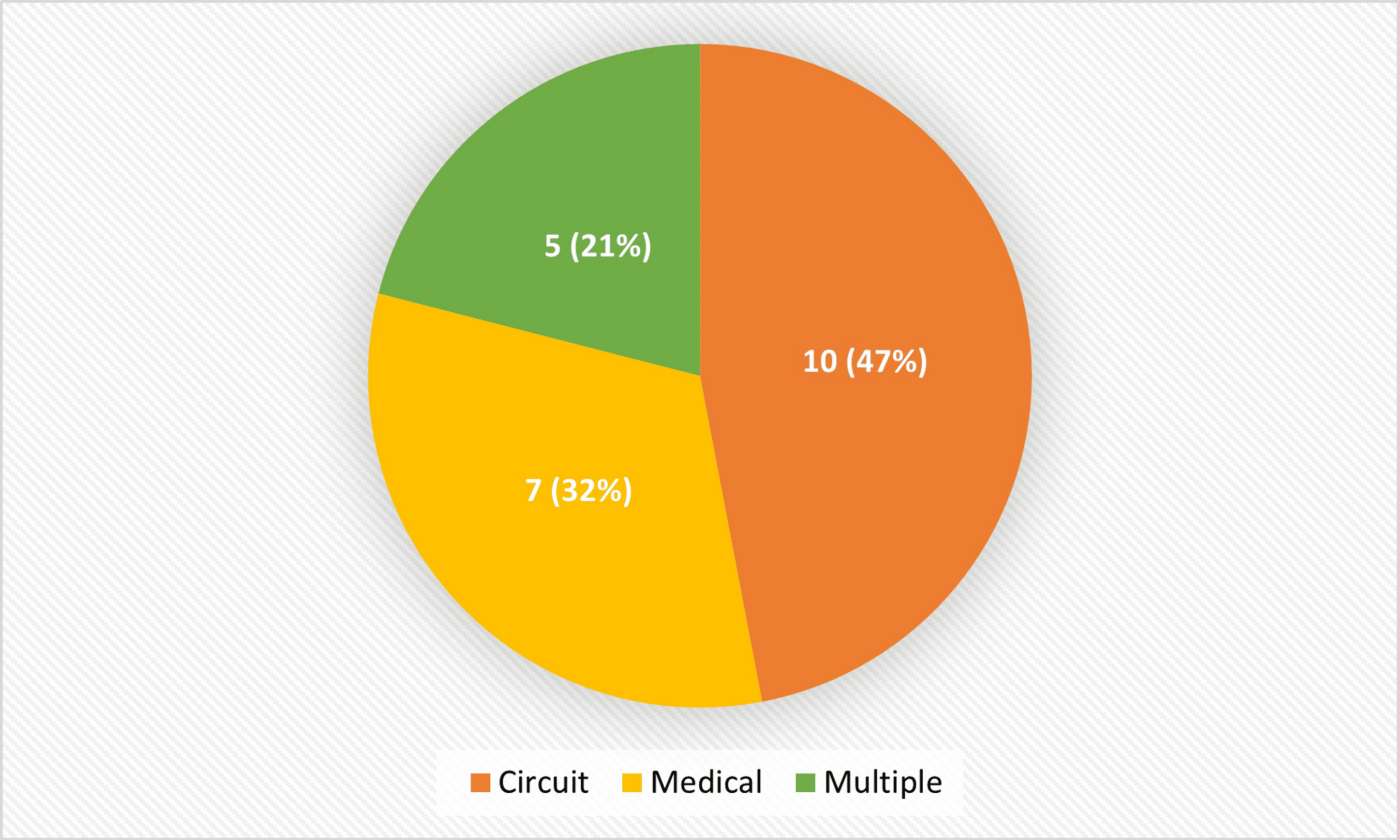
Distribution of reported complications Created by the authors

Reported complications were closely associated with the type of platform used and the cannulation technique employed. The most frequently observed issues, as summarized in Table 4 and Figure 4, included cannulation failure, defined as either the inability to establish umbilical vascular access at the start of the procedure or early loss of circuit patency after initial cannulation, reported in six studies; circuit thrombosis in four studies; bleeding or increased risk of intracranial hemorrhage in four studies; infection or sepsis in three studies; and hemodynamic instability in five studies.

**Table 4:** Reported complications across included studies

No	Complication	Studies Reporting (n)
1	Cannulation failure	6
2	Circuit thrombosis	4
3	Bleeding / IVH concern	4
4	Infection / Sepsis	3
5	Hemodynamic instability	5

Later-generation studies that incorporated nitric oxide-releasing coatings and refined EVE system configurations demonstrated a reduction in infection rates and bleeding events, indicating progressive improvements in safety and biocompatibility over time. These complication trends link directly to the methodological limitations' objective and highlight areas requiring further innovation before human trials can be considered.

Risk of Bias Assessment

**Figure 5:**
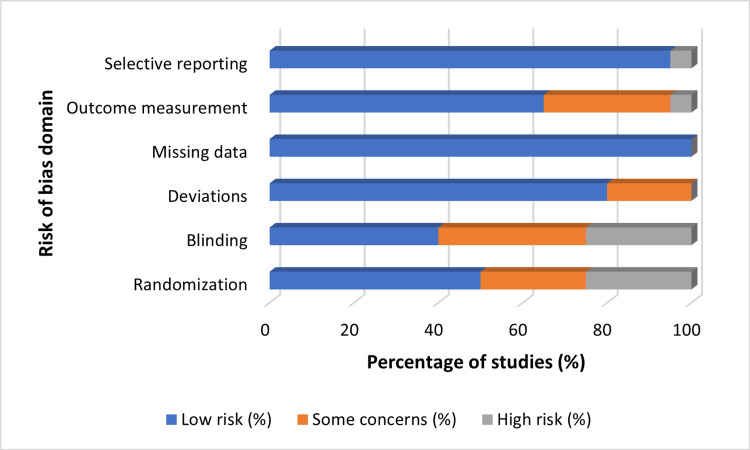
Risk-of-bias summary across studies Created by the authors • Selective reporting: Low risk = 20 (91%), Some concerns = 2 (9%), High risk = 0 (0%) • Outcome measurement: Low risk = 16 (73%), Some concerns = 5 (23%), High risk = 1 (4%) • Missing data: Low risk = 18 (82%), Some concerns = 3 (14%), High risk = 1 (4%) • Deviations from intended interventions: Low risk = 14 (64%), Some concerns = 6 (27%), High risk = 2 (9%) • Blinding: Low risk = 10 (45%), Some concerns = 7 (32%), High risk = 5 (23%) • Randomization: Low risk = 8 (36%), Some concerns = 9 (41%), High risk = 5 (23%)

Using a SYRCLE-adapted framework [20], the risk of bias was assessed across five domains, as shown in Table 5 and Figure 5. 

**Table 5:** Summary of risk-of-bias domains using SYRCLE criteria across 22 preclinical AWT studies AWT: artificial womb technology

No	Bias Domain	Low Risk (n)	High/Unclear Risk (n)
1	Selection Bias	10	12
2	Performance Bias	8	14
3	Detection Bias	9	13
4	Attrition Bias	14	8
5	Reporting Bias	16	6

Selection bias was reported as low in 10 studies and high or unclear in 12, while performance bias was low in 8 studies and high or unclear in 14. Detection bias was low in 9 studies and high or unclear in 13, attrition bias was low in 14 studies and high or unclear in 8, and reporting bias was low in 16 studies and high or unclear in 6. More recent studies exhibited enhanced methodological transparency, including the use of predefined outcome measures and well-defined comparator groups, which contributed to reducing concerns related to reporting bias. These findings emphasize the methodological variability that must be considered when interpreting feasibility and organ-level results.

Discussion

Over the past decade, AWT has progressed from experimental short-duration trials to demonstrating sustained extracorporeal support with meaningful organ-protective potential. Early studies achieved only 24-70 hours of survival in pump-driven VV circuits [13], but subsequent refinements, including improved oxygenator performance, optimized flow resistance, and more consistent anticoagulation practices, enabled support periods of approximately one week using VV and EVE systems.

In 2017, the feasibility of maintaining fetal lambs for 28 days using a pumpless AV biobag system was demonstrated, reflecting an important conceptual advance in partial ectogestation [14]. Across these platforms, fetal circulation remained uninterrupted, ductus arteriosus patency was preserved, and gas-exchange parameters generally stayed within physiologic ranges [15]. Some studies also documented increases in bone length and body weight, suggesting that AWT can support both survival and somatic development during critical periviable stages [16,17]. Taken together, these observations extend beyond mere feasibility and highlight the developmental support potential of AWT under controlled experimental conditions.

Organ-Level Interpretation

Across included experiments, multiple organ systems demonstrated encouraging outcomes. Pulmonary findings showed reduced injury markers and evidence of progressive alveolarization in models supported for 10-14 days [18], while neurological assessments consistently demonstrated maintained cerebral oxygenation and preserved structural integrity. Gastrointestinal and splenic maturation were also observed, albeit with occasional mild histological injury [19]. These cross-organ findings support the hypothesis, introduced in the Introduction, that a fluid-filled environment without mechanical ventilation may offer substantial organ protection during periviable development.

Nonetheless, translation remains constrained by technical and physiological complications. Cannulation failure, thrombosis, bleeding risk, infection, and hemodynamic instability remained common challenges, particularly in early studies [20]. Later-generation systems incorporating nitric-oxide-releasing surfaces reduced thrombosis and infection, enabling week-long runs without systemic anticoagulation [21]. These complications mirror known issues in neonatal ECMO, suggesting that experience from human extracorporeal support may provide useful translational guidance.

Methodological Considerations

Methodological quality varied across studies. Performance and detection bias were frequently high due to absent blinding and randomization, while selection bias was inconsistently reported. Attrition and reporting bias were generally lower, with most studies clearly documenting outcomes. The heterogeneity of study design, outcome measurement, and circuit configuration underscores the need for standardized reporting frameworks before meaningful cross-study comparison or meta-analysis can be attempted.

Ethical and Regulatory Interpretation

As AWT advances toward potential early-phase clinical evaluation, ethical considerations become central. Unlike conventional NICU patients, recipients of AWT would be fetuses in a transitional gestational state, requiring re-examination of legal, moral, and clinical definitions of the patient [22]. Prospective consent would likely need to occur prenatally, with reaffirmation after delivery, ensuring that families clearly understand the transitional fetal status and investigational nature of AWT.

Robust safety protocols, including predefined stopping rules, emergency rescue pathways, and long-term follow-up, will be necessary to ensure ethical oversight [22]. Equity also emerged as a critical concern, with none of the included studies originating from low- or middle-income countries (LMICs) [24]. This geographic imbalance suggests a risk of widening disparities if AWT becomes clinically viable without intentional global planning. Regulatory agencies, such as the U.S. FDA, have begun issuing preliminary guidance, emphasizing harmonized outcome measures, standardized trial designs, and strengthened consent models [25].

Integrated Synthesis and Translational Implications

Synthesizing the evidence across platforms, AWT demonstrates increasing stability, improved biocompatibility, and encouraging organ-level protection. Pulmonary and neurological outcomes are particularly promising, while hematologic and infection-related complications remain ongoing challenges. Although these findings provide a rationale for progressing toward staged first-in-human evaluation, the timeline for translation remains uncertain and will depend on the safety, reproducibility, and ethical viability of early feasibility studies. Deliberate international collaboration, especially with LMIC representation, will be essential to ensure equitable global access, avoid technological disparities, and promote responsible integration of AWT into neonatal care frameworks.

## Conclusions

Preclinical research on artificial womb technology (AWT) has progressed beyond proof-of-concept, demonstrating the ability to sustain fetal support, maintain physiological stability, and provide early indications of organ protection. Advances in circuit design, the development of anticoagulation-free surfaces, and continued platform refinement have contributed to reducing complications and improving overall outcomes. If successfully translated into clinical settings, AWT has the potential to redefine the limits of viability and reshape neonatal intensive care for infants born at 22-24 weeks of gestation; however, the timeline for any clinical application remains uncertain and will depend on the outcomes of future phased safety and feasibility trials. This review consolidates a decade of preclinical AWT research, emphasizing platform diversity, organ-level outcomes, and translational relevance. Its primary strength lies in synthesizing data from large-animal studies alongside evolving ethical frameworks. However, the field still faces several limitations, including small sample sizes, methodological heterogeneity, and variability in reporting quality, all of which preclude meta-analysis and limit the generalizability of findings to human neonates. Future research should focus on standardizing outcome measures, improving methodological rigor through enhanced blinding and randomization, and prioritizing the development of anticoagulation-free platforms. Early human trials must be conducted with strong ethical guidance, a focus on safety, and a commitment to global inclusivity to ensure equitable access to the potential benefits of AWT.
